# Prognostic value of neutrophils to lymphocytes and platelets ratio for 28-day mortality in patients with acute respiratory distress syndrome: a retrospective study

**DOI:** 10.1186/s12890-022-02112-w

**Published:** 2022-08-15

**Authors:** Shiyu Nie, Hongjin Wang, Qiuyu Liu, Ze Tang, Wu Tao, Nian Wang

**Affiliations:** Department of Critical Care Medicine, Yongchuan Hospital, Chongqing Medical University, No. 439 Xuanhua Road, Yongchuan District, Chongqing, 402160 China

**Keywords:** Acute respiratory distress syndrome, 28-day mortality, Neutrophils to lymphocytes and platelets ratio, Neutrophil-to-lymphocyte ratio, Neutrophil count, Lymphocyte count, Platelet count

## Abstract

**Background:**

Acute respiratory distress syndrome (ARDS) is a rapidly progressive and fatal respiratory failure disease that often occurs in critically ill patients. Since ARDS is associated with immune dysregulation and coagulation abnormalities, it is necessary to identify an appropriate predictor that can accurately predict ARDS mortality based on its pathophysiology. Therefore, this study aimed to evaluate the clinical value of neutrophils to lymphocytes and platelets ratio (N/LPR) in predicting 28-day mortality in ARDS patients.

**Methods:**

From July 2018 to October 2021, the medical records of ARDS patients were retrospective reviewed. Neutrophil count, lymphocyte count, and platelet count were collected, and the neutrophil-to-lymphocyte ratio (NLR) and N/LPR were calculated. Multivariate logistic regression analyses were performed to identify independent predictors of 28-day mortality in ARDS. Receiver operating characteristic (ROC) curve with the area under curve (AUC) was used to evaluate optimal cut-off values for 28-day mortality in ARDS. Kaplan–Meier analysis was used to estimate the 28-day survival probabilities stratified by optimal cut-off values of N/LPR and NLR.

**Results:**

A total of 136 ARDS patients were included in this study and were further divided into survivors (*n* = 69) and non-survivors (*n* = 67) groups according to their survival status on day 28. There were no significant differences between the two groups in age, sex, history of smoking and drinking, comorbidities, and reasons of admission (*P* > 0.05). Non-survivors had significantly higher neutrophil counts, NLR and N/LPR and had significantly lower platelet counts than survivors (*P* < 0.05). Multivariate regression analysis revealed that N/LPR, NLR and platelet counts were independent predictors for 28-day mortality in ARDS (*P* < 0.05). The ROC analyses showed that N/LPR with optimal cut-off value of 10.57 (sensitivity: 74.6%; specificity: 72.5%) is a more reliable predictor for 28-day mortality in ARDS than NLR and platelet count (AUC: 0.785 vs. 0.679 vs. 0.326). Further subgroup analysis confirmed that ARDS patients with N/LPR < 10.57 had significantly lower 28-day mortality than patients with N/LPR ≥ 10.57 (*P* < 0.001). Kaplan–Meier analysis also confirmed that ARDS patients with N/LPR < 10.57 had significantly longer survival.

**Conclusion:**

N/LPR is an independent risk factor associated with 28-day mortality in ARDS patients and shows better performance in predicting mortality rate than NLR.

## Background

Acute respiratory distress syndrome (ARDS) is a common cause of respiratory failure, especially in life-threating patients in the intensive care unit (ICU) [1, 2]. Despite recent advances in medical treatment and understanding of the pathophysiology of ARDS, its high morbidity and mortality continue to severely impact patient health and has become a substantial public health burden [3, 4]. In a large observational study of ICU patients in 50 countries, the prevalence of ARDS in ICU admission was 10.4%, with a very high in-hospital mortality rate of 35–46% [5]. Therefore, it is necessary and important to identify crucial prognostic factors for ARDS patients to provide timely early intervention to improve survival.

The neutrophil-to-lymphocyte ratio (NLR) is the number of neutrophils divided by the number of lymphocytes measured in peripheral blood. The clinical value of NLR has been extensively studied and considered as a reliable and emerging predictor reflecting the relationship the immune system and diseases [6]. The attractive characteristics of NLR is that it can be rapidly obtained and calculated from peripheral blood, and the value can reflect the status of innate immunity and adaptive immunity through neutrophils and lymphocytes, respectively. An elevated NLR value usually means an increase in neutrophils (reflecting a proinflammatory state) and/or a decrease in lymphocytes (reflecting the weak adaptive immunity to diseases). Therefore, NLR has been suggested to predict the severity and mortality of various diseases, such as sepsis [7], community-acquired pneumonia [8, 9], chronic obstructive pulmonary disease [10, 11], acute limb ischemia [12], acute cholecystitis [13], cardiovascular disease [14–16], and intracerebral hemorrhage [17, 18], as well as the recent pandemic COVID-19 pneumonia [14, 19–21].

Although several studies also reported the predictive prognostic value of NLR in ARDS patients [22–24], NLR only reflects the inflammatory and immune status in these patients. In fact, the occurrence and development of ARDS is more complex, which is related to the crosstalk between systemic inflammatory response, abnormal immune regulation, and coagulation dysfunction [25]. Given the high mortality rate of ARDS, there is an urgent need to identify novel predictive biomarkers or improve current predictors to more precisely predict the prognosis of ARDS patients. In the recent years, the neutrophils to lymphocytes and platelets ratio (N/LPR) has received considerable attention. Several recent studies reveal the prognostic value of N/LPR in assessing systemic inflammatory responses and demonstrate that N/LPR can reflect the tight interplay between inflammation, coagulation, and immune response [26, 27]. Given the importance of platelets in ARDS pathogenesis [28], we speculate that N/LPR may be a more appropriate prognostic indicator for predicting mortality of ARDS patients. Therefore, this study aimed to investigate whether N/LPR could be used to predict the 28-day mortality in ARDS patients and further compare it with NLR indicator.

## Materials and methods

### Patient population

From July 2018 to October 2021, medical records of ARDS patients from Department of Critical Care Medicine of Yongchuan Hospital affiliated to Chongqing Medical University were retrospectively reviewed. The inclusion criteria were as follows: (1) Age 18 or over; (2) Confirmed diagnosis of ARDS according to the guideline for diagnosis and therapy of acute lung injury and acute respiratory distress syndrome [29]; (3) Stable hemodynamics; and (4) Complete medical history. The exclusion criteria were as follows: (1) Pregnant and lactating women; (2) Hematologic disorders; (3) Malignant tumors and/or immune disorder; and (4) Taking drugs within the past week that may affect the number of neutrophils, lymphocytes and platelets. This study was approved by the Institutional Review Board (IRB) of Chongqing Medical University (IRB number: #2020–84) and conducted in accordance with the Declaration of Helsinki. The requirement of written informed consent was waived by the IRB due to the retrospective nature of the study.

### Study design

The study endpoint was the 28-day mortality. Clinical data of all eligible patients were collected, including age, sex, a history of smoking and drinking, comorbidities, and reason of admission. Acute physiology and chronic health evaluation II (APACHE-II) score, sequential organ failure assessment (SOFA) score and laboratory test results (PaO2/FiO2 ratio, neutrophil counts, lymphocyte counts, and platelet counts) were obtained within 24 h after the diagnosis of ARDS. The NLR is calculated as the ratio between the neutrophil and lymphocyte counts measured in peripheral blood, as follows: Neutrophil count / lymphocyte count. The N/LPR is calculated as follows: Neutrophil count × 100 / (lymphocyte count × platelet count). If multiple values were evaluated within 24 h after diagnosis, the worst value was used. All eligible patients were followed up for 28 days to calculate the 28-day mortality.

### Statistical analysis

All statistical analyses in this study were performed using SPSS version 28 (IBM Corp, Armonk, NY). Continuous data with normal distributions were presented with mean ± standard deviation (SD), and the difference between groups was analyzed using t-test. Continuous data with probability distributions were presented with medians and quartiles, and the difference between groups was analyzed Mann–Whitney U test. Categories data were presented with frequencies and percentage and compared using Chi-square test. Binary logistic regression analysis was used to identify potential predictors associated with the 28-days mortality in ARDS patients. The predictive prognostic values of each risk factors for mortality were evaluated by the receiver operating characteristic (ROC) curve method and area under the curve (AUC). The predictive ability between N/LPR, NLR and platelet count were compared using DeLong’s method [30]. Stratified analysis of 28-day mortality of ARDS was performed using the optimal cut-off values. Survival curves were estimated by Kaplan–Meier survival analysis, and the differences between groups were determined by log-rank test. A *P*-value < 0.05 was considered statistically significant.

## Results

### Baseline characteristics of ARDS patients

**Fig. 1:**
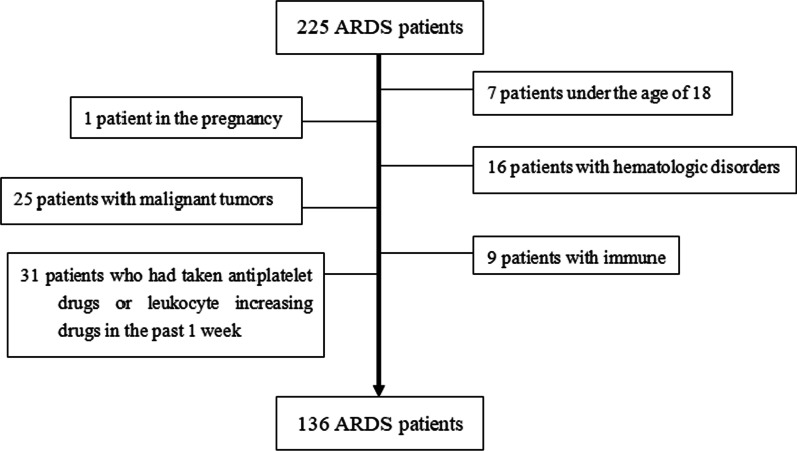
Flow-chart of the patient inclusion process. *Abbreviations*: ARDS, acute respiratory distress syndrome

A total of 225 patients diagnosed with ARDS were screened. Among them, 89 patients were excluded from this study, including 7 patients under the age of 18, 1 patient in the pregnancy, 16 patients with hematologic disorders, 25 patients with malignant tumors, 9 patients with immune disorders, and 31 patients who had taken antiplatelet drugs or leukocyte increasing drugs in the past 1 week. Finally, a total of 136 eligible ARDS patients were included in the analysis (Fig. 1). Among them, 67 ARDS patients died within 28 days (assigned to the non-survivors group), while 69 survived (assigned to the survivors group). Table 1 shows the baseline and clinical characteristics of the survivors and non-survivors. There were no significant differences between the two groups with respect to age, sex, smoking history, drinking history, comorbidities, reason of admission, and days of mechanical ventilation (*P* > 0.05). Compared with non-survivors, survivors had significantly lower APACHE-II scores (22 vs. 27, *P* = 0.026) and SOFA scores (7 vs. 9, *P* < 0.001) and significantly higher PaO2/FiO2 ratios (173.55 vs. 147.64, *P* = 0.011) at admission.

**Table 1:** Baseline characteristics of ARDS patients according to the 28-day survival status

Variables	Survivors (*n* = 69)	Non-survivors (*n* = 67)	*P*-value
Age, years	61.8 ± 14.211	65.1 ± 12.553	0.157
Male	42 (60.9%)	49 (73.1%)	0.129
Smoking	29 (42. 0%)	38 (56.7%)	0.087
Alcohol	27 (39.1%)	34 (50.7%)	0.173
Comorbidities			0.183
Hypertension	27 (39.1%)	18 (26.9%)	
Diabetes mellitus	7 (10.1%)	4 (6.0%)	
Coronary artery disease	0 (0%)	3 (4.5%)	
Reason of admission			0.608
Severe pneumonia	36 (52.2%)	43 (64.2%)	
Non-pulmonary sepsis	5 (7.2%)	3 (4.5%)	
Trauma	5 (7.2%)	3 (4.5%)	
Severe acute pancreatitis	9 (13.0%)	6 (9.0%)	
Poisoning	7 (10.1%)	4 (6.0%)	
Aspiration	3 (4.3%)	4 (6.0%)	
Pulmonary embolism	3 (4.3%)	2 (3.0%)	
DIC	0 (0.0%)	2 (3.0%)	
Burns	1 (1.4%)	0 (0.0%)	
Mechanical ventilation, day	5 (1.5–8.0)	4 (2.0–7.0)	0.885
APACHE-II score	22 (20.0–26.5)	27 (20.0–30.0)	**0.026**
SOFA score	7 (5.0–9.0)	9 (7.0–11.0)	** < 0.001**
PaO2/FiO2	173.55 ± 61.108	147.64 ± 55.167	**0.011**

Data are presented as median (IQR) or number (%). Bold indicates factors that are statistically significant

*DIC* disseminated intravascular coagulation; *APACHE-II* acute physiology and chronic health evaluation II; *SOFA* sequential organ failure assessment; *IQR* interquartile range; *ARDS* acute respiratory distress syndrome; *FiO2* Fraction of inspired oxygen; *PaO2* Partial pressure of arterial oxygen

Table 2 shows the laboratory parameters of ARDS patients on admission. There were statistically significant differences between groups in neutrophil count, platelet count, NLR and N/LPR (*P* < 0.05). Non-survivors had significantly higher neutrophil counts, NLR and N/LPR and lower platelet counts than survivors (all *P* < 0.05). Moreover, there was no significant difference between groups in the lymphocyte counts (*P* > 0.05).

**Table 2:** Laboratory parameters of ARDS patients according to the 28-day survival status

Variables	Survivors (*n* = 69)	Non-survivors (*n* = 67)	*P*-value
Neutrophil count (10^9^/L)	9.14	11.52	**0.001**
Lymphocyte count (10^9^/L)	0.69	0.64	0.519
Platelet count (10^9^/L)	167	125	**0.000**
NLR	8.25	15.02	**0.000**
N/LPR	12.48	16.92	**0.000**

Bold indicates factors that are statistically significant

*NLR* neutrophil-to-lymphocyte ratio; *N/LPR* neutrophils to lymphocytes and platelets ratio; *ARDS* acute respiratory distress syndrome

### Identification of N/LRP as an independent predictor for 28-day mortality in ARDS patients

**Fig. 2:**
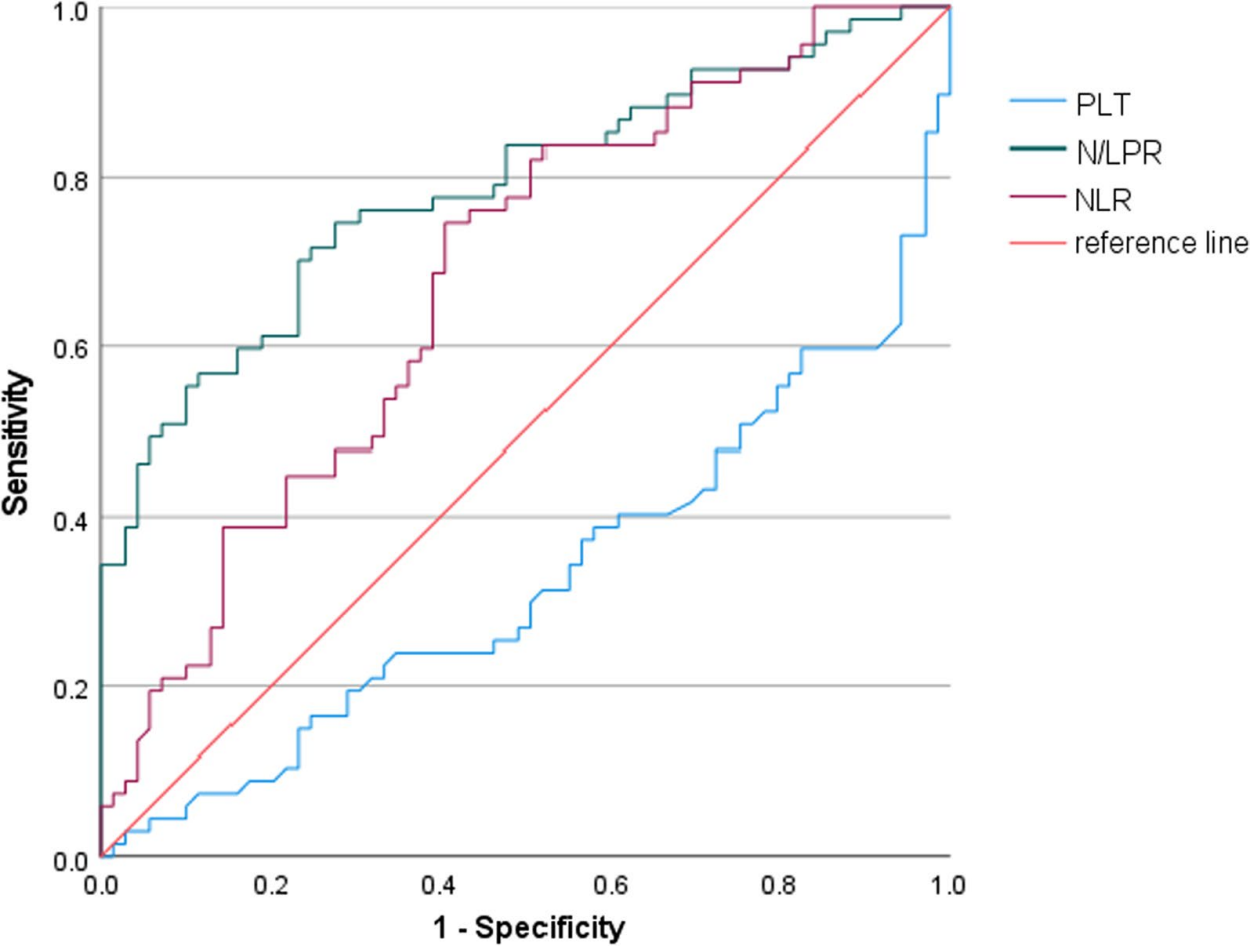
ROC curves for predicting 28-day mortality in ARDS patients. *Abbreviations*: N/LPR, neutrophils to lymphocytes and platelets ratio; NLR, neutrophil-to-lymphocyte ratio; PLT, Platelet count; ROC, receiver operating characteristic; ARDS, acute respiratory distress syndrome

Univariate and multivariate logistic regression analyses were performed to identify independent predictor of 28-day mortality in ARDS patients. As shown in Table 3, N/LPR (OR = 8.934, *P* = 0.003), NLR (OR = 5.218, *P* = 0.022) and platelet counts (OR = 3.895, *P* = 0.048) were independent predictors associated with 28-day mortality in ARDS patients. Therefore, ROC curve and Youden index analyses were further performed to assess the predictive performance of the three predictors and to identify their optimal cut-off values. As shown in Fig. 2, N/LPR showed the largest area under the curve, followed by NLR and then platelet count. In addition, comparisons of the ROC curves of N/LPR, NLR and platelet count further showed that AUC of N/LPR was significantly higher than the AUC of NLR (*P* = 0.006) and platelet count (*P* = 0.009), suggesting that N/LPR had the best predictive values in 28-day mortality than NLR and platelet count. Table 4 showed the best cut-off value of N/LPR, NLR and platelet count established by the Youden index. The AUCs of the N/LPR, NLR and platelet count were 0.785 (95% CI: 0.708–0.862, *P* = 0. 000), 0.679 (95% CI: 0.589–0.768, *P* = 0. 000) and 0.326 (95% CI: 0.235–0.417, *P* = 0.000), respectively. When the optimal cut-off value of N/LPR was 10.57 to discriminate the 28-day mortality in ARDS patients, the sensitivity and specificity were 74.6% and 72.5%, respectively. The optimal cut-off value for NLR was 14.20, with a sensitivity of 74.6% and a specificity of 59.4%.

**Table 3:** Binary logistic regression analysis of 28-day mortality prediction for ARDS patients

Variables	B	Walds	OR	*P*-value
APACHE-II	0.04	0.047	0.699	0.403
PaO2/FiO2	− 0.004	0.004	1.16	0.282
SOFA	0.007	0.121	0.003	0.954
Neutrophil count	0.103	0.053	3.694	0.055
Platelet count	0.018	0.009	3.895	**0.048**
N/LPR	0.475	0.159	8.934	**0.003**
NLR	− 0.204	0.089	5.218	**0.022**

Bold indicates factors that are statistically significant

*APACHE-II* acute physiology and chronic health evaluation II; *SOFA* sequential organ failure assessment; *NLR* neutrophil-to-lymphocyte ratio; *N/LPR* neutrophils to lymphocytes and platelets ratio; *ARDS* acute respiratory distress syndrome; *OR* Odds ratio, *FiO2* Fraction of inspired oxygen; *PaO2* Partial pressure of arterial oxygen

**Table 4:** Predictive value of potential indicators in predicting 28-day mortality in ARDS patients

Indicators	AUC	95% CI	Optimal cutoff value	Sensitivity (%)	Specificity (%)
Platelet count	0.326	0.235–0.417	–	–	–
N/LPR	0.785	0.708–0.862	10.57	74.6	72.5
NLR	0.679	0.589–0.768	14.20	74.6	59.4

*NLR* neutrophil-to-lymphocyte ratio; *N/LPR* neutrophils to lymphocytes and platelets ratio; *ARDS* acute respiratory distress syndrome; AUC, Area under the curve; *CI* Confidence interval

### Validation of the efficacy of N/LPR with an optimal cutoff value in predicting 28-day mortality in ARDS patients

**Fig. 3:**
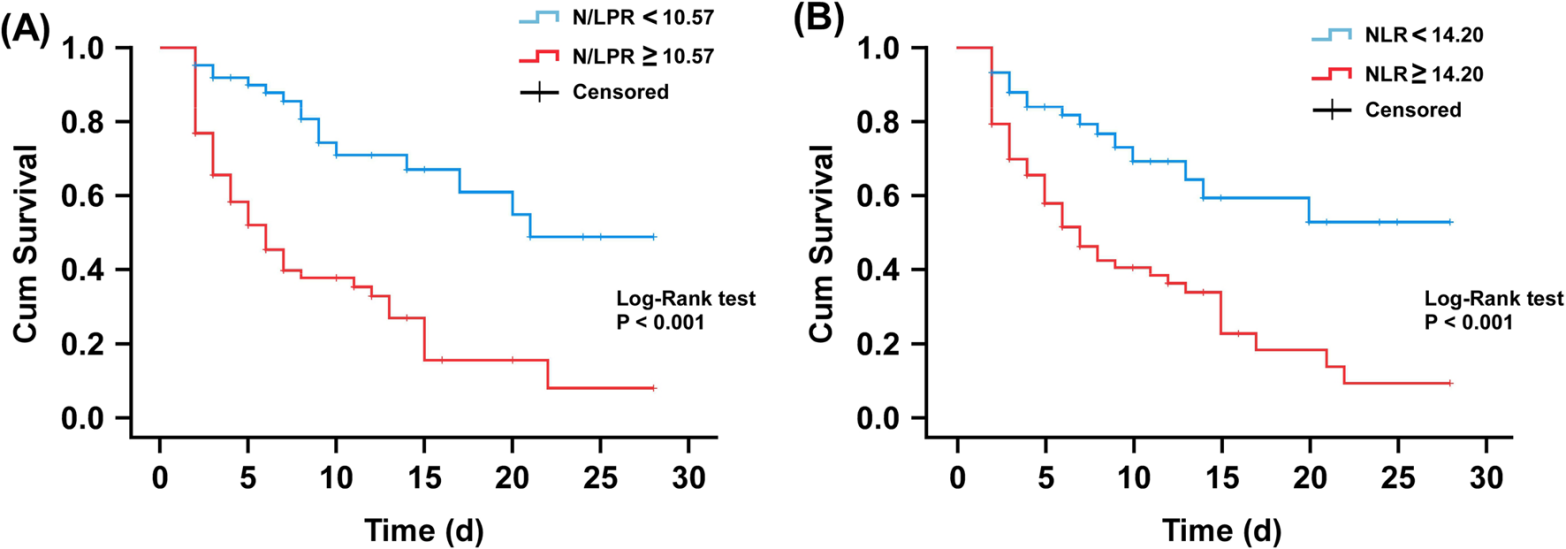
Kaplan–Meier curves for 28-day mortality of ARDS patients with different peripheral blood N/LPR and NLR. **A** N/LPR with an optimal cut-off value of 10.57. **B** ARDS patients with an optimal cut-off value of 14.20. *Abbreviations*: N/LPR, neutrophils to lymphocytes and platelets ratio, NLR, neutrophil-to-lymphocyte ratio, ARDS, acute respiratory distress syndrome

To further confirm the predictive values of N/LPR and NLR for 28-day mortality, 136 ARDS patients were divided into two groups according to the optimal cutoff values determined by above ROC analysis. The number of deaths, 28-day mortality and mean survival time in the two groups were subsequently calculated. As shown in Table 5, ARDS patients with N/LPR ≥ 10.57 had higher number of death than ARDS patients with N/LPR < 10.57 (50 vs. 17). In addition, ARDS patients with N/LPR ≥ 10.57 had significantly higher 28-day mortality (68.5% vs. 27.0%, *P* < 0.001) and significantly shorter survival time (5 vs. 8 months, *P* = 0.002). Regarding NLR, the number of death and 28-day mortality in ARDS patients with NLR ≥ 14.20 were notably higher than that in ARDS patients with NLR < 14.20 (Table 5). ARDS patients with higher NLR also survived significantly longer than those with lower NLR (*P* = 0.048). Furthermore, the predictive values of N/LPR and NLR were further validated using the Kaplan–Meier analysis. The results showed that the 28-day mortality of ARDS patients with N/LPR ≥ 10.57 was significantly lower than that of patients with N/LPR < 10.57 (28-day mortality: 68.5% vs. 27.0%, *P* < 0.001, Fig. 3A). Meanwhile, patients with an NLR ≥ 14.20 also had significantly worse survival probability than patients with NLR < 14.20 (28-day mortality: 64.9% vs. 28.8%, *P* < 0.001, Fig. 3B). The results of these analyses suggest that both N/LPR and NLR are good predictors of 28-day mortality in ARDS patients.

**Table 5:** Comparison of 28-day mortality and survival in ARDS patients with different N/LPR and NLR values

	Patients	Death (*n*)	28-day mortality (%)	Median survival day (IQR)
N/LPR < 10.57	63	17	27.0	8 (3–14)
N/LPR ≥ 10.57	73	50	68.5	5 (2–9)
*P*-value			** < 0.001**	**0.002**
NLR < 14.20	59	17	28.8	8 (3–12)
NLR ≥ 14.20	77	50	64.9	5 (2.5–10.5)
*P*-value			** < 0.001**	**0.048**

Bold indicates factors that are statistically significant

*N/LPR* neutrophils to lymphocytes and platelets ratio, *NLR* neutrophil-to-lymphocyte ratio, *SD* Standard deviation, *ARDS* acute respiratory distress syndrome

## Discussion

N/LPR has received increasing attention in the recent years, because it can not only present the balance between systemic inflammation and immunity, but also additionally reflect coagulation abnormality in acute illness [26, 27, 31]. Given the critical role of platelets in lung injury development and resolution and the appreciable in-hospital mortality of ARDS [28, 32, 33], this study aimed to evaluate the predictive values of NLR and N/LPR in ARDS patients. This study demonstrates that NLR is a moderate predictor of 28-day mortality in ARDS, whereas platelet count is a poor predictor. Moreover, the addition of platelets to NLR setting, the N/LPR predictor, further improved the predictive value of 28-day mortality in ARDS. The optimal cut-off value of N/LPR was identified as 10.57, with an acceptable sensitivity of 74.6% and specificity of 72.5%. ARDS patients with elevated N/LPR was associated with increased risk of 28-day in-hospital mortality. Survival curve analyses further confirmed that the predictor N/LPR was superior to NLR and platelet count in predicting in-hospital mortality of ARDS patients. Therefore, the N/LPR value may assist with the prediction of the high-risk mortality of ARDS patients, providing a reference for limited intensive care resources in the overloaded medical system.

The clinical predictive value of N/LPR was first identified by Koo et al. in a retrospective study of 1099 patients who underwent cardiovascular surgeries [26]. Results indicated that higher N/LPR was associated with increased risk of acute kidney injury and 5-year mortality in patients after high-risk cardiovascular surgeries. Furthermore, N/LPR showed significantly better predictive ability than NLR and platelet count. Another retrospective analysis by Liu et al. also confirmed the clinical value of N/LPR in reflecting inflammatory response, immune function and coagulation dysfunction of sepsis patients [31]. N/LPR with a cut-off value of 10.65 (with a sensitivity of 75% and a specificity of 85%) has better predictive value than NLR in predicting 28-day mortality of sepsis patients admitted to ICU. Consistently, the results of this study not only support the superiority of N/LPR, but also further extends the applicability of N/LPR for predicting the in-hospital mortality in high-risk ARDS patients. For ARDS patients, N/LPR was superior to NLR and platelet count in predicting 28-day mortality, because N/LPR had the highest AUC (0.785) and acceptable sensitivity (74.6%) and specificity (72.5%) when the optimal cut-off value was set at 10.57. Although NLR also exhibited a comparable sensitivity of 74.6% and a similar Kaplan–Meier curves (Fig. 3) to N/LPR, both the AUC (0.679) and specificity (59.4%) of NLR were worse than that of N/LPR.

The superiority of N/LPR predictors in ARDS patients may be attributed to the addition of platelet parameters. ARDS is a life-threatening pulmonary syndrome manifested by a series of pathophysiologic processes, such as alveolar flooding, hypoxemia, inflammatory dysregulation, lung deformation, and intravascular coagulation, as well as platelet activation [28, 34, 35]. Platelets and coagulation mutually influence each other, and their close interplay contributes to the balance of hemostasis and bleeding [36]. Therefore, the association between platelets and ARDS may be due to the extensive cross talk between coagulation and inflammation [37–39]: platelets interact with neutrophils to form platelet–neutrophil complexes, which recruit more neutrophils, trigger endothelial and immune cell activation, and finally the development of ARDS. In our study, non-survivors had significantly lower platelet counts than survivors, suggesting that low platelet counts are associated with poor prognosis in ARDS. This finding is also supported by several studies showing that platelet count is an independent predictor of mortality in ARDS patients [38, 40, 41]. Furthermore, our findings are consistent with the study by Wang et al. [42] that thrombocytopenia accelerate the progression of ARDS and increase mortality in critically ill patients.

Although ARDS is also one of the common clinical manifestations of severe COVID-19, the role of coagulation in ARDS caused by COVID-19 is also a matter of debate. Thromboembolic manifestations are generally considered to be associated with increased mortality and morbidity in COVID-19 patients [43]. In the recent study by Wu et al., coagulation dysfunction was identified as a risk factor associated with the development of ARDS and subsequent progression to death in patients with COVID-19 pneumonia [44]. Coagulation dysfunction in patients with ARDS is mainly due to the exposure and interaction of tissue factor (TF) with neutrophil elastase, resulting in the activation of coagulation cascade [25, 37]. In the prospective study by Ozolina et al. [40], patients who developed ARDS had significantly higher plasma TF than patients who did not develop ARDS. Therefore, neutrophil elastase inhibitors have been reported to improve the prognosis of ARDS associated with sever sepsis [45] and are also recommended for management of ARDS with coagulopathy caused by COVID-19 [46]. However, a recent study by Yin et al. found that patients with severe pneumonia with COVID-19 infection actually have higher platelet counts than that without COVID-19 infection [47]. In addition, some COVID-19 patients do have mild thrombocytopenia, but disseminated intravascular coagulopathy and severe bleeding events (common reasons for ARDS development) are uncommon in COVID-19 patients [47, 48]. However, there were no COVID-19 patients in our study population. Therefore, the predictive value of N/LPR may not currently be applicable to ARDS caused by severe COVID-19. Nevertheless, a recent study of COVID-19 patients by Regolo et al. [21] revealed the superior predictive value of NLR in predicting mortality and worse outcomes in COVID-19 patients, suggesting that NLR may be useful in predicting worsening respiratory failure in Covid-19 patients. Future studies are needed to test and compare the predictive value of N/LPR and NLR in COVID-19-related ARDS.

There were several limitations in this study. The ARDS population in this study is rather small, and the data obtained in China population cannot be extrapolated to other populations. Therefore, the applicability of N/LPR to other populations or races need to be further explored. The small sample size also limits our further exploration of other potential confounding factors that may affect the prognosis of ARDS. In addition, since there were no patients with COVID-19 in our study population, the predictive value of N/LPR may not currently be applicable for ARDS patients caused by COVID-19. Future large-scale prospective studies should be conducted to overcome the current disadvantages of this study and to further confirm the clinical application of N/LPR predictor for 28-day mortality in ARDS patients.

## Conclusions

Our study revealed the clinical values of N/LPR in ARDS patients. N/LPR with a cutoff value of 10.57 is not only a good predictor of 28-day mortality in ARDS patients, but also shows better predicting accuracy than NLR. Therefore, N/LPR should be considered as one of the routine indicators for monitoring and reporting the health status of ARDS patients in clinical practice, which can provide judgment reference for physicians.

## Data Availability

The data that support the findings of this study are available from the corresponding author upon reasonable request.

## Abbreviations

ARDS: Acute respiratory distress syndrome
N/LPR: Neutrophils to lymphocytes and platelets ratio
NLR: Neutrophil-to-lymphocyte ratio
ROC: Receiver operating characteristic
APACHE-II: Acute physiology and chronic health evaluation II
SOFA: Sequential organ failure assessment
AUC: Area under the curve
CI: Confidence interval
ICU: Intensive care unit
IRB: Institutional review board
SD: Standard deviation
OR: Odds ratio
COVID-19: Coronavirus disease 2019
TF: Tissue factor
ALI: Acute lung injury
FiO2: Fraction of inspired oxygen
PaO2: Partial pressure of arterial oxygen
IQR: Interquartile range
